# An unusual association between focal segmental sclerosis and lupus nephritis: a distinct concept from lupus podocytopathy?

**DOI:** 10.1007/s13730-014-0142-1

**Published:** 2014-08-17

**Authors:** Hironari Hanaoka, Akinori Hashiguchi, Konosuke Konishi, Masataka Kuwana, Tsutomu Takeuchi

**Affiliations:** 1 Division of Rheumatology, Department of Internal Medicine, Keio University School of Medicine, 35 Shinanomachi, Shinjuku-ku, Tokyo, 160-8582 Japan; 2 Department of Pathology, Keio University School of Medicine, Tokyo, Japan; 3 Division of Endocrinology, Metabolism and Nephrology, Department of Internal Medicine, Keio University School of Medicine, Tokyo, Japan

**Keywords:** Lupus nephritis, Lupus podocytopathy, Focal segmental sclerosis

## Abstract

Lupus nephritis (LN) is usually associated with immune deposition in the glomerular capillary wall. On the other hand, focal segmental glomerulosclerosis (FSGS) is not typically associated with immune deposition, and its pathogenesis includes podocyte damage and loss. The definition of lupus podocytopathy (LP) excludes patients with electron-dense glomerular basement membrane deposits. Here, we report the case of an LN patient with nephrotic proteinuria. Renal pathology demonstrated focal endocapillary hypercellularity superimposed on foam cells. Immunofluorescence revealed diffuse global subepithelial immune deposits, and electron microscopy showed electron-dense glomerular basement membrane deposits and diffuse foot process effacement. Treatment with steroid and cyclosporine improved her proteinuria. Post-treatment renal re-biopsy revealed focal segmental sclerotic lesions closely resembling FSGS. These results indicate that the pathogenesis of this case may involve an FSGS-like condition or podocytopathic change. It is possible that careful examination would reveal podocytopathic changes other than LP in patients previously diagnosed as LN class III + V. Further investigations are needed to understand FSGS-like pathological changes accompanied with capillary immune deposits in LN.

## Introduction

Systemic lupus erythematosus (SLE) is an autoimmune disease characterized by the production of autoantibodies to various cellular components [1]. Approximately 50 % of patients with SLE exhibit lupus nephritis (LN) [2]. The American Society of Rheumatology recently published guidelines for LN screening, treatment, and management, which recommended renal biopsy for pathological classification of all patients presenting clinical evidence of LN [3].

LN is usually associated with immune aggregate deposition in the glomerular capillary wall, frequently accompanied by endocapillary proliferations and necrosis [2]. Focal segmental glomerulosclerosis (FSGS) is histologically defined by the segmental obliteration of glomerular capillaries by extracellular matrix, and is a cause of idiopathic nephrotic syndrome and end-stage renal disease. FSGS does not involve granular immune-electron-dense deposits, and immunofluorescence typically reveals coarse segmental staining for IgM and C3 entrapped in the area of hyalinosis. FSGS is a common histopathological lesion that can represent a primary podocytopathy. Barisoni et al. [4] defied podocytopathy characterized clinically by the presence of nephrotic syndrome in diseases that are caused by abnormal glomerular cell functions which begin with podocyte injury or dysfunction. In this report, podocytopathies are defined by four patterns of glomerular lesions: minimal change disease, FSGS, diffuse mesangial sclerosis and collapsing glomerulopathy. However, many proteinuric diseases, including viral infections, metabolic syndrome and immune complex deposition or in situ formation (IgA nephropathy, membranous nephropathy and LN), can cause podocyte injury [4]. Thus SLE-related podocytopathy or podocyte injury may contain 2 pathological conditions with or without immune deposits. The latter is so-called lupus podocytopathy (LP) [5]. The pathogenesis of SLE-related podocytopathy still remains unclear.

Recent reports introduce a concept of LP, with a definition that excludes patients with immune deposition on capillary wall [5]. Until now, coexistence of FSGS-like lesions and immune deposits on capillary in SLE patient had not been described.

Here we describe an SLE patient with nephrotic proteinuria, whose renal pathology revealed FSGS-like findings with capillary immune deposits. Treatment with steroid and cyclosporine led to a good clinical response. This case indicates that podocytopathic changes may be involved in the pathogenesis, suggesting a direction for further investigation in patients with LN class III + V.

## Case report

In April 2011, an 81-year-old woman with a history of photosensitivity and hypertension was admitted to our hospital due to bilateral edema of the legs and nephrotic proteinuria (7.0 g/day). Her proteinuria was not detected before March 2011 by screening test by the family doctor. At admission, her height was 153 cm, weight was 40 kg, and blood pressure was 138/70 mmHg with treatment of candesartan 8 mg/day. Physical examination showed bilateral pitting edema of her legs and palpable superficial inguinal lymphadenopathies.

Laboratory results were as follows: serum creatinine 1.27 mg/dL; sodium 138.8 mEq/L; potassium 4.5 mEq/L; hemoglobin 14.6 g/dL; white blood cells 12,300/μL; platelet count 42,000/μL; serum total proteins 7.4 g/dL; and albumin 2.1 g/dL; total cholesterol 224 mg/dl, low-density lipoprotein cholesterol 119 mg/dL, high-density lipoprotein cholesterol 60 mg/dL, triglyceride 96 mg/dL. She had an antinuclear antibody (ANA) titer of 1:40, with a speckled pattern that was associated with the presence of anti-dsDNA antibody (28 IU/mL). Tests were negative for anti-Sm, anti-RNP, anti-SS-B, anti-SS-A, and anti-cardiolipin IgG antibodies. Her CH50 level was low (14.3 U/mL). Microscopic urinalysis showed 51–100 red blood cells and 3–5 white blood cells per hyper field. Proteinuria was 9.3 g/day and selectivity index was 0.01. Chest and abdominal CT revealed no abnormalities. She was diagnosed with SLE based on thrombocytopenia, proteinuria, positive ANA with anti-dsDNA antibody, and history of photosensitivity.

**Fig. 1:**
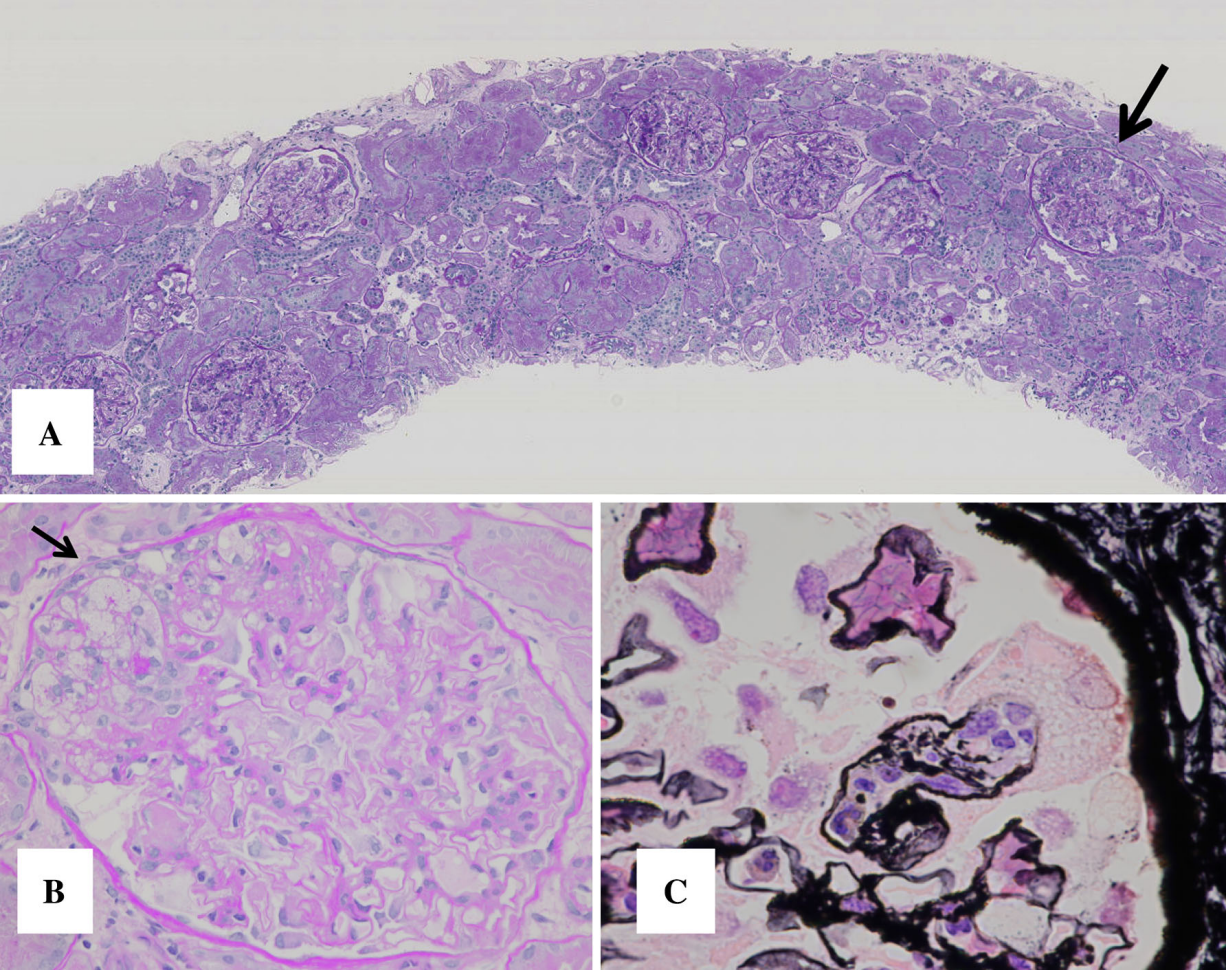
Renal biopsy before treatment for light microscopy. **a** The specimen contained 26 glomeruli; 8 exhibited global sclerosis, 2 revealed focal endocapillary hypercellularity superimposed on several foam cells (*arrow*). **b** PAS staining revealed focal segmental sclerotic lesions with foam cells (*arrow*). **c** PASM staining showed a glomerulus exhibiting segmental capillary collapse with podocyte hypetrophy

Before renal biopsy, we decided to treat her by prednisolone (PSL) 40 mg/day to normalize platelet count. Five days after the treatment, platelet count increased to 127,000/μL. Renal biopsy was performed 7 days after the treatment. The specimen for light microscopy contained 26 glomeruli; 8 exhibited global sclerosis, 2 revealed focal endocapillary hypercellularity superimposed on several foam cells (Fig. 1).

**Fig. 2:**
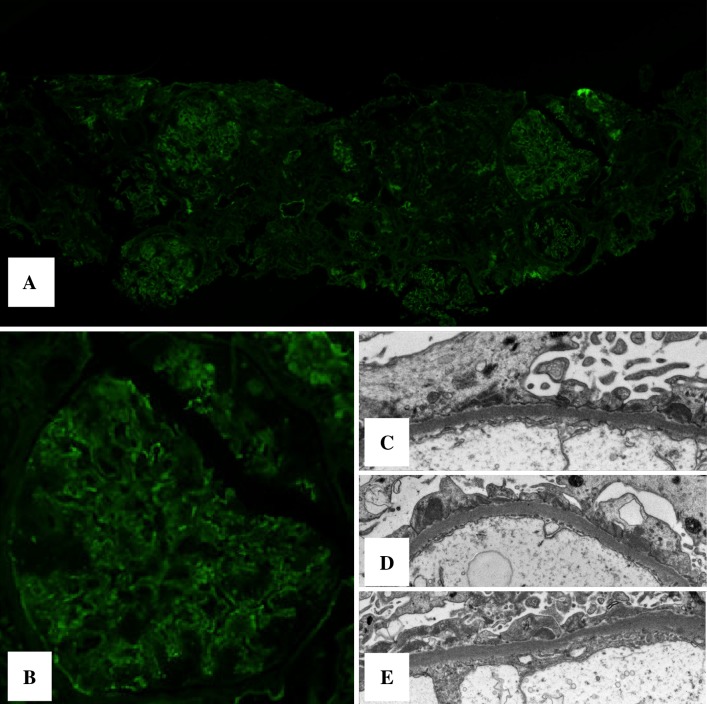
Renal biopsy before treatment for immunofluorescence and electron microscopy. **a** Immunofluorescence with anti-IgG (FITC-anti-human) showed weak capillary staining (extracted from whole slide image). **b** Magnification of a representative affected glomerulus (FITC-anti-human; extracted from whole slide image). **c**–**e** Electron microscopy revealed diffuse foot process effacement and sparse subepithelial deposits

Immunofluorescence (IF) showed low-intensity diffuse global granular capillary staining of IgG, IgA, IgM, C3, and C1q. Analysis by electron microscopy (EM) revealed electron-dense glomerular basement membrane deposits and foot process effacement (Fig. 2).

**Fig. 3:**
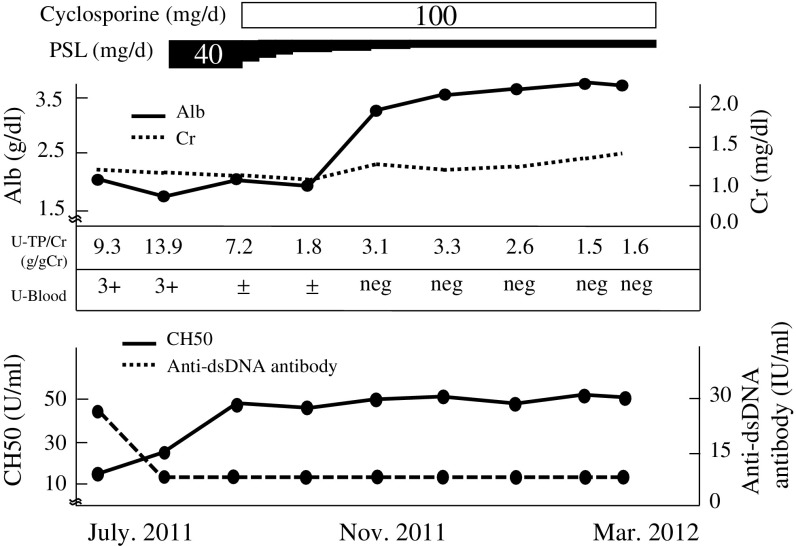
Patient’s clinical course. Proteinuria, serum albumin, and CH50 levels improved after administration of PSL and cyclosporine. After 7 months of therapy, her proteinuria decreased to 10 % of the pre-treatment level. *PSL* prednisolone, *Alb* albumin, *Cr* creatinine, *U-TP/Cr* urinary protein:urinary creatinine ratio, *U-Blood* urinary occult blood

**Fig. 4:**
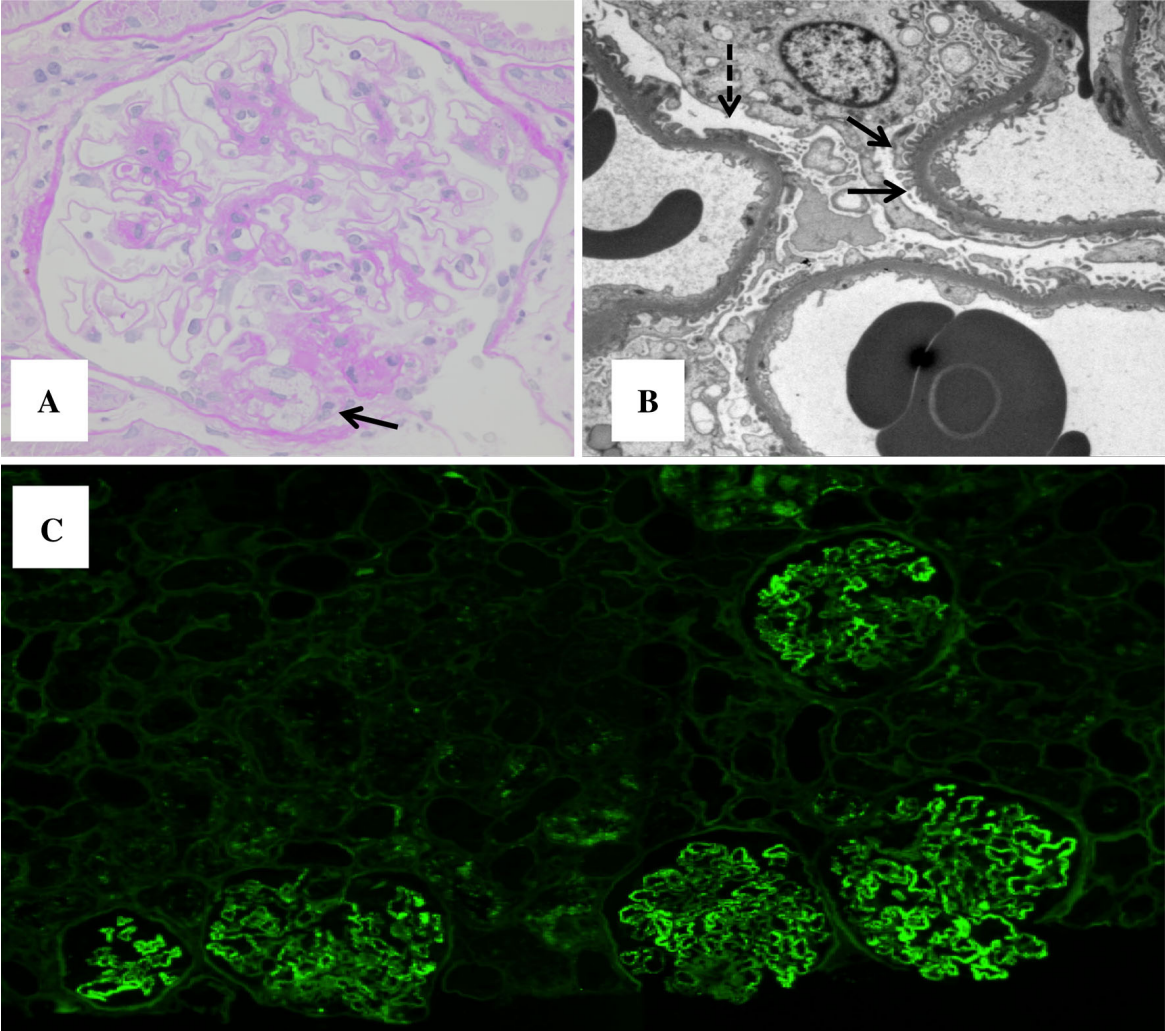
Renal re-biopsy after 7 months of treatment with glucocorticoid and cyclosporine. **a** Light microscopy with PAS staining showed a segmental sclerosed glomerulus and endocapillary foam cells (*arrow*). **b** Electron microscopy showed segmental subepithelial deposit (*dotted arrow*), and foot process effacement (*filled arrow*). **c** Immunofluorescence still detected abundant IgG deposits in the capillary wall (FITC-anti-human; extracted from whole slide image)

This patient would have been diagnosed as LN class III (A) + V or III (A) because of scanty subepithelial immune deposition; however, we observed focal segmental foam cells closely resembling the cellular variant of FSGS. Based on experience with FSGS from July 2011, we added cyclosporine 100 mg/day on the initiated treatment with PSL. Within 3 months, the patient’s proteinuria rapidly improved from 9.3 to 1.8 g/day, and her leg edema completely disappeared. Her CH50 level gradually increased, and her anti-dsDNA antibody titer decreased below the normal limit (Fig. 3). In March 2012, we performed a kidney re-biopsy. We observed focal segmental sclerotic lesions and endocapillary foam cells. IF with IgG, C3, and C1q showed bright granular staining, and EM showed electron-dense deposits at the same site. Diffuse foot process effacement was still present (Fig. 4).

## Discussion

Here, we describe the interesting case of an SLE patient with FSGS-like lesions accompanied by capillary immune deposits. Steroid and cyclosporine therapy led to a good clinical response, such that only a small amount of proteinuria is now detectable.

Podocytes may play a main role in the pathological process of FSGS [6]. Podocyte injury leads to effacement of podocyte foot processes, which is the major structural correlate of nephrotic proteinuria in FSGS. A classification of histological variants recognizes not-otherwise-specified (NOS), perihilar, cellular, tip, and collapsing diseases. The cellular variant is characterized by expansile segmental lesions with endocapillary hypercellularity, often including foam cells and infiltrating leukocytes, with variable glomerular epithelial cell hyperplasia [7]. Primary FSGS is initially treated with a high dose of glucocorticoid, typically 1 mg per kg of body weight daily. In adults, a response to glucocorticoids may take up to 16 weeks [8]. Glucocorticoid-resistant FSGS is treated with a calcineurin inhibitor, either cyclosporine or tacrolimus. These agents take 4–6 months to induce remission. It has been demonstrated that calcineurin inhibitors have a direct effect on podocytes by stabilizing the actin cytoskeleton [9].

The notion of podocyte-targeted nephropathy in nephritic LN was recently introduced. Based on pathological findings, Kraft et al. [5] proposed the new concept of LP, defined as including normal observations by light microscopy, and mesangial proliferative glomerulonephritis or FSGS lesions in SLE patients with nephrotic syndrome. This study excluded biopsies that showed endocapillary proliferation or necrosis by light microscopy, or electron-dense glomerular basement membrane deposits by electron microscopy. The onset of nephrotic syndrome correlated with SLE onset in patients who satisfied the LP criteria. The authors finally concluded that LP is the result of active SLE.

Our case would have been classified as LN class III (A) + V or III (A), except that pathological observations included focal segmental foam cells, closely resembling cellular variant FSGS. The post-treatment renal pathology showed focal segmental sclerosis, suggestive of FSGS-like lesions. In spite of the good clinical response, a higher intensity of subepithelial immune deposits was observed after treatment. These results indicate that podocyte injury without involvement of immune complex may play a pathogenic role in the patient’s proteinuria.

According to the original proposal of pathologic classification of FSGS, D’Agati et al. [10] described that focal and segmental scaring following other primary glomerular diseases, such as lupus nephritis, should be ruled out by immunofluorescence and electron microscopy. So, careful observation is needed for diagnosing SLE patients with FSGS-like morphology. FSGS-like lesions in patients with SLE are reported pretty rare. Huong et al. [11] retrospectively analyzed 175 SLE patients who underwent at least 1 renal biopsy from 1980 to 1993 in France. By light microscopic analysis, 2 patients (1.1 %) had FSGS, one with normal urinalysis or serum creatinine, and the other with abnormal urinalysis or elevated serum creatinine levels. Hertig et al. [12] described 11 SLE patients with idiopathic nephrotic syndrome. FSGS-like lesions were identified in seven patients, and only one of them was positive for immunoglobulins. Moreover, the patient showed the presence of IgG, IgM, C3 and C1q deposits confined to the mesangium. However, our present case did not coincide with the area where immune deposits exist, and may thus represent a new category of SLE-related podocytopathy.

In summary, here we reported a case of nephrotic LN with FSGS-like pathological changes accompanied by capillary immune deposits. It is possible that this condition may exist in other patients who were previously diagnosed as LN class III + V or III (A). Careful observation may be needed for the glomerular lesion with scanty subepithelial deposits in patient with nephritic syndrome to identify the overlapping mechanisms of the podocytopathy.
